# Preliminary evidence that Bunyamwera virus causes severe disease characterized by systemic vascular and multiorgan necrosis in an immunocompromised mouse model

**DOI:** 10.1099/jgv.0.002040

**Published:** 2024-11-07

**Authors:** M. Fausta Dutuze, Samantha D. Clark, Fabio Del Piero, Rebecca C. Christofferson

**Affiliations:** 1 Rwanda Institute for Conservation Agriculture, Kigali, Rwanda; 2 Department of Pathobiological Sciences, School of Veterinary Medicine, Louisiana State University, Baton Rouge, Louisiana 70803, USA

**Keywords:** Bunyamwera, Bunyavirus, mouse model, Orthobunyavirus, pathogenesis model

## Abstract

Bunyamwera virus (BUNV) is the prototypical member of the Bunyamwera serogroup within the *Orthobunyvirus* genus. BUNV is transmitted by mosquito vectors of the genera *Culex*, *Aedes* and *Anopheles* and has historically circulated in East Africa, though the transmission has been observed in Argentina. BUNV has been identified as an agent of human and animal disease and has also been misdiagnosed as other agents. BUNV is often thought to be an agent of mild febrile illness in humans, though it can cause abortions in ruminants and neurological disease in horses. Joint pain and gastritis have also been attributed to BUNV. There are limited data concerning the possible spectrum of disease and extent of pathogenesis of BUNV infection, and there are currently no therapeutics or vaccines available. Furthermore, options for animal models for Orthobunyaviruses in general – of which BUNV is the prototypical member – are limited. Eight mice deficient in the type I interferon response were infected with BUNV, and all developed overt disease. All mice developed detectable viraemia and clinical signs, including weight loss, hunched posture and lethargy. Three of the eight mice developed severe diseases, including vascular necrosis and necrosis in the liver, lungs, reproductive organs, bone marrow and spleen, as well as haemorrhages (*n*=1) and severe diffuse facial oedema (*n*=3), reminiscent of the pathology of Schmallenberg and the Arenaviruses Lassa and Lujo viruses. Thus, BUNV infection of IRF3/7 DKO mice could serve as a BSL-2 model for severe diseases of higher-risk group viruses, which often must be studied at BSL-4. Additionally, our results suggest that BUNV may have the ability to cause severe disease in immunocompromised hosts. Thus, further investigation into the potential spectrum of pathogenesis due to BUNV is important to prioritize for outbreak response, diagnostics and the development of countermeasures.

## Introduction

Bunyamwera virus (BUNV) is the prototypical member of the Bunyamwera serogroup within the *Orthobunyvirus* genus in the family Peribunyaviridae [1]. BUNV is transmitted by mosquito vectors of the genera *Culex*, *Aedes* and *Anopheles* [2,3] and has mostly been observed in East Africa, though transmission has been observed in Argentina [4,5]. BUNV has been identified as an agent of human and animal disease and has also been misdiagnosed as other agents [4,6,9]. This is likely due in part to the fact that molecular-based techniques, such as nucleic acid amplification and sequencing, are not always performed; diagnosis is based on clinical manifestations, and surveillance for Orthobunyaviruses in general – including BUNV – is very nearly absent. For example, in an outbreak of the disease in Sudan, infection with NRIV – a reassortant of BUNV – was misdiagnosed as malaria [10]. Furthermore, in Rwanda, clinically presumed Rift Valley fever cases in cattle were subsequently determined to be BUNV in 3% of cases [11]. Therefore, the burden of disease caused by BUNV is likely underreported, and its public health impact is underappreciated.

BUNV is often thought to be an agent of mild febrile illness in humans, though it can cause abortions in ruminants and neurological disease in horses [4,7, 12]. Joint pain and gastritis have also been attributed to BUNV [7]. Closely related members of the Bunyamwera serogroup, such as Cache Valley fever virus, Batai virus and Ngari virus, have been associated with more severe disease in humans, including neurological disease, arthralgia and haemorrhagic fever [13,17]. As BUNV is understudied, there is a question of whether we understand the extent of its ability to cause disease, as other African-origin viruses (e.g. Zika) have been associated with novel sequelae upon large outbreaks [18,19].

There are some data to suggest that BUNV might be capable of causing more disease that is currently attributed to infection. For example, studies showed that suckling (2-day-old) ICR Swiss mice inoculated intracerebrally with high doses of BUNV (10^10^ LD50 ml^−1^) developed severe encephalitis and died with peak viraemia at 16 h post-inoculation [20]. Another study showed that high doses of BUNV (10^9^ p.f.u. ml^−1^) inoculated intraperitoneally in Swiss albino mice resulted in neurological symptoms, such as limb paralysis, tremors and disorientation in suckling mice (SM) between 2 and 6 days, while 6-week-old mice showed no signs of disease [21]. Furthermore, a study in non-human primates demonstrated a spectrum of diseases, including death during the viraemic period [22]. Together, these studies suggest that a spectrum of disease is possible in mammals, but whether these observations represent the extent of pathogenesis is unclear. There are currently no therapeutics or vaccines available for BUNV, and understanding the potential spectrum of pathogenesis due to BUNV is important to prioritize the development of such.

## Methods

### Viruses

Viral stocks used in this research project were BUNV strain 6547-8, with a titre of 6.65×10^6^ p.f.u. ml^−1^, obtained from the World Reference Center for Emerging Viruses and Arboviruses at the University of Texas Medical Branch (Galveston, TX). This strain of BUNV was originally isolated from *Aedes* spp. in Uganda in 1943. Prior to our use, the passage history for this virus was SM 47/Vero 2, SM3/Vero 2 and SM4/baby hamster kidney cells (BHK) 1/Vero 2. In our hands, it was passaged twice more in Vero cells prior to our experiments. Before the actual infection experiment, a single mouse was subcutaneously inoculated with 100 µl of 6.65×10^5^ p.f.u. ml^−1^ for an infection test. The mouse died at 4 dpi, and thus, we decreased the dose to 10^3^ p.f.u. ml^−1^ to achieve a non-uniformly fatal model.

### Mice

Eight female IRF3/7^-/- -/-^ double knock-out mice were used for this study, aged 8–9 weeks at the time of infection. Mice were subcutaneously inoculated with 100 µl of 6.65×10^3^ p.f.u. ml^−1^ of BUNV. They were weighed every day until 6 dpi and bled every day until 4 dpi via cheek bleed. Mice could not be bled after 4 dpi because of severe vascular lesions and because the maximum sampling blood volume had been reached (or nearly so). Mice were checked and weighed daily according to IACUC protocol 12-079.

### Sample processing

Blood samples were incubated for 30 min at room temperature and serum was separated and collected after centrifugation (6000 relative centrifugal force, 4 min, 4 °C) and stored at −80 °C until viral RNA extraction and amplification. RNA extraction was performed using the KingFisher (Thermo Fisher) automated extraction platform (according to the instructions of the manufacturer) as previously described. Viral RNA was detected by reverse transcription quantitative real time (RT-qRT) PCR using the SuperScript III One-Step RT-PCR System with Platinum Taq DNA Polymerase (Life Technologies) on a Roche LightCycler 480 (Roche). We used primers and probes targeting the M segments [11,23, 24].

### Statistics

Kaplan–Meier analysis was used to determine the timing of clinical sign development and mortality, as well as median time to mortality. Viraemia (p.f.u. ml^−1^) and weight (in grams) are presented as mean titre per day post-infection with sd. Weight loss was calculated as the difference in weight on a particular day compared to the weight of that same mouse at day 0. Percent weight loss was calculated as the difference in weight on days 1–6 divided by the weight of that mouse at day 0. Means and sd are graphed to visualize trends. Differences in metrics over days were tested via Kruskal–Wallis analysis of variance. Post-hoc test for pairwise differences was performed using Dunn’s test.

### Pathology

After death, all mice were grossly examined. The organs of three mice were placed in 10% neutral buffered formalin and sent to the Louisiana State University Animal Disease Diagnostic Laboratory (LADDL–LSUDX) for histopathology. Tissues were dehydrated in alcohol and xylene and embedded in paraffin. Five-micrometre-thick tissue sections were then obtained for slide preparation before being stained with haematoxylin and eosin and cover slipped. Afterwards, the slides were examined via light microscopy, and pathological changes were recorded by the American College of Veterinary Pathologists board-certified pathologists of the LSU School of Veterinary Medicine.

## Results

### Infection kinetics of BUNV in IRF 3/7 DKO mice

**Fig. 1.**
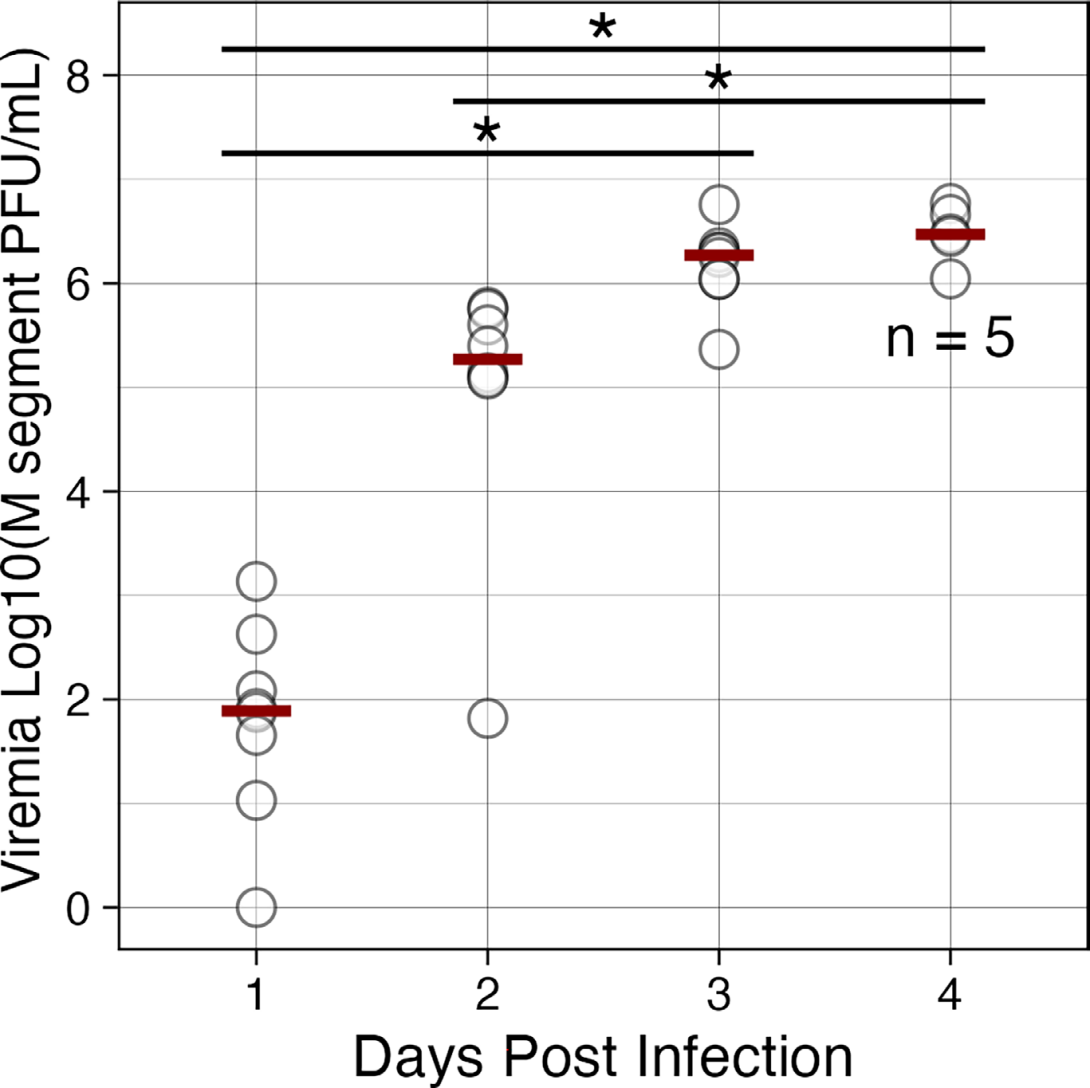
BUNV viraemia in IRF 3/7 DKO Mice. Viraemia of 8 mice (days 1–3) and 5 mice (day 4) for BUNV. Red lines indicate median values.

Most (7/8) mice developed viraemia by day 1 post-subcutaneous inoculation, and viraemia increased through day 4 post-infection (Fig. 1). Only one mouse (ID 384) did not have detectable viraemia at day 1 though this mouse developed high viraemia by day 3, similar to the other mice (Fig. S1, available in the online version of this article). Viraemia differed across the 4 days tested (*P*<0.0001). Pairwise comparisons demonstrated that while there was an observable increase in viraemia from days 1 to 2, this was not significant (*P*=0.1771). Day 1 was statistically different from day 3 (*P*=0.0009) and day 4 (*P*=0.0002). Day 2 was statistically different from day 4 (*P*=0.0291), which showed the highest observed median viraemia of 6.47 Log_10_ genome equivalents per ml, though this was only slightly elevated from a median of 6.27 Log_10_ genome equivalents per ml on day 3 post-infection. The maximum viraemia values were observed on day 4 in mice 381 and 382 (Fig. S1).

### Pathogenesis of BUNV infection in IRF 3/7 DKO mice

**Fig. 2.**
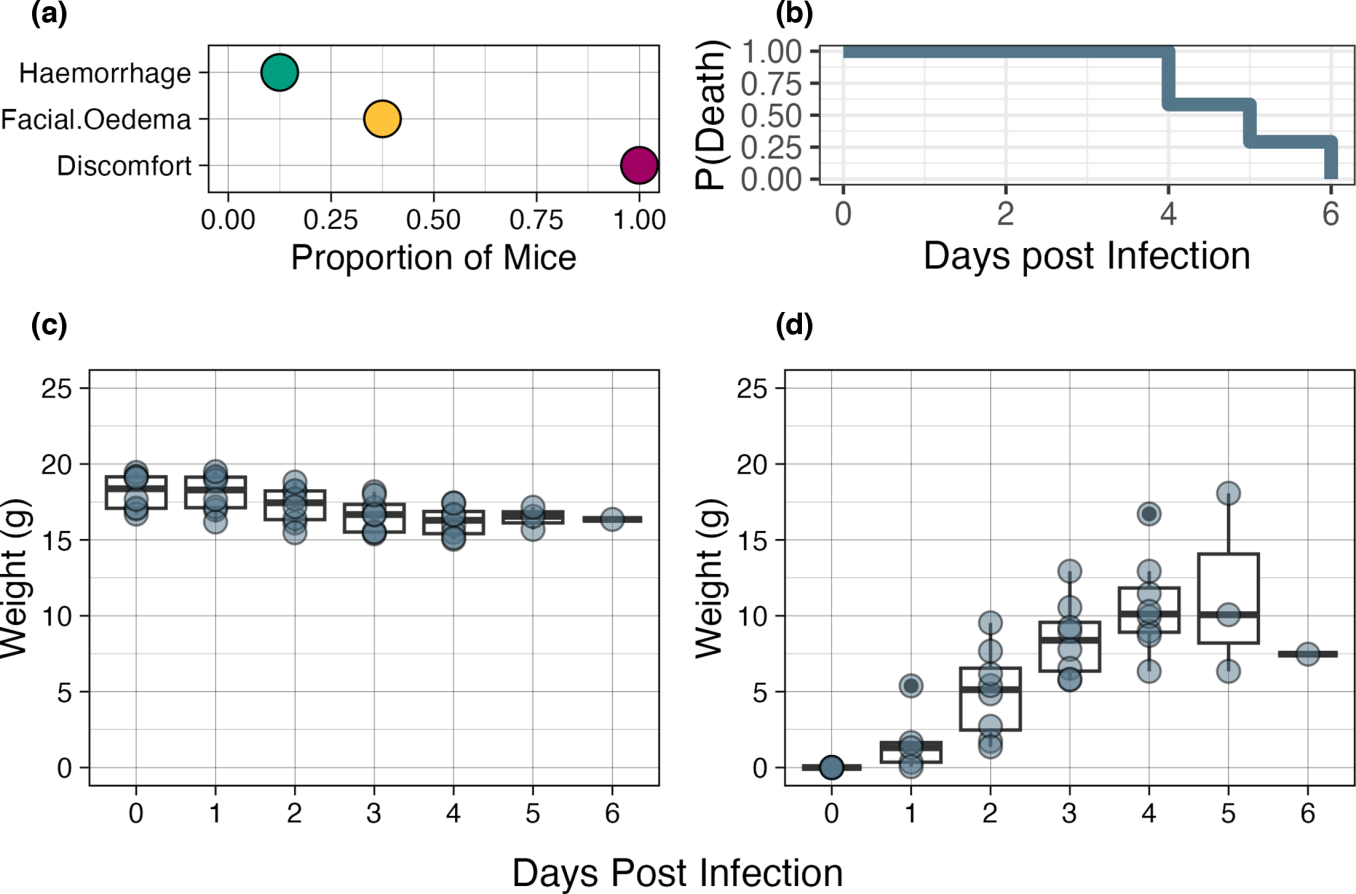
Clinical course of BUNV infection in IRF 3/7 DKO mice. (a) The proportion of eight mice that developed clinical signs. (**b**) Kaplan–Meier analysis for time to death, with a median time to death of 5 days and 100% mortality by 6 days post-infection. Median weight (**c**) and percent change in weight (**d**) across all 6 observed days. *N*=8 for all days unless otherwise noted.

All mice developed clinical signs of discomfort, including ruffled fur and a hunched posture (Fig. 2a).There was 100% mortality by day 6, with the median time to death at 5 days post-inoculation (Fig. 2b). All mice lost weight (Figs 2c, d and S2). Weight loss steadily increased through day 4 for all eight mice, while additional weight loss was observed for the three surviving mice on day 5 post-infection (Fig. 2c, d). We noted that there was significant haemorrhaging in the abdominal cavity upon dissection of one mouse (#378), which died at 5 days post-exposure. Interestingly, this mouse did not have the highest viraemia or weight loss.

### Histologic lesions associated with severe BUNV-induced disease

**Fig. 3.**
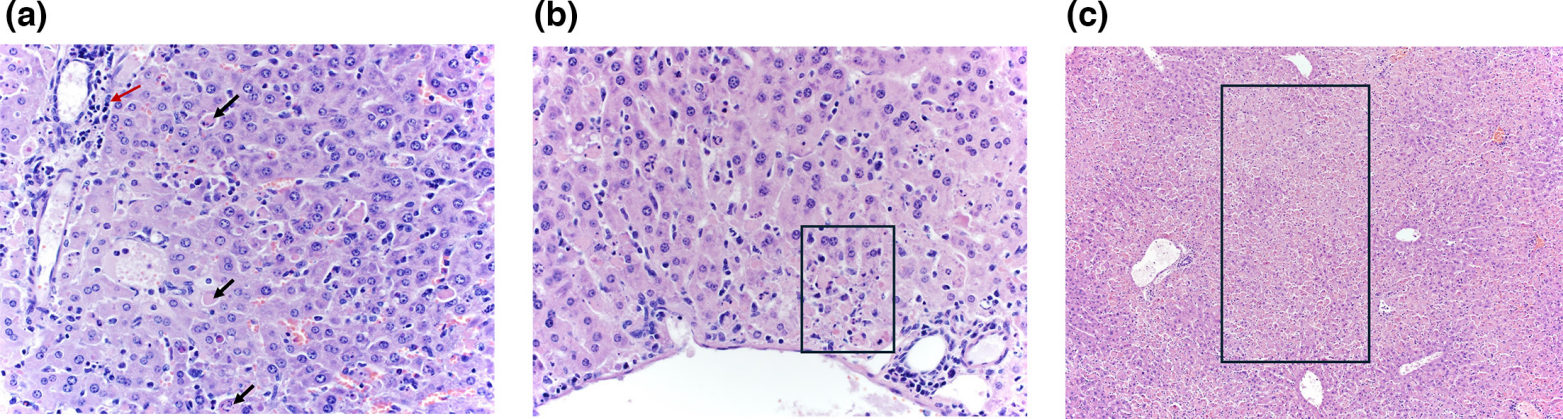
Histologic lesions of the liver of BUNV-infected IRF3/7 DKO mice. (a) Liver single-cell necrosis and portal hepatitis (mouse 381, 40×). Black arrows indicate multifocal single-cell necrosis, while red arrow indicates portal hepatitis. (b) Liver periportal area necrosis of the hepatocellular limiting plate (mouse 381, 40×). (c) Severe random, acute coagulative necrosis in the liver (mouse 382, 10×).

BUNV disease in IRF 3/7 DKO mice was multisystemic, causing vascular necrosis and necrosis in the liver, lungs, spleen, bone marrow, reproductive system (uterus and ovary) and skin. The liver was affected with multifocal single-cell necrosis, ample areas or periportal coagulative necrosis (Fig. 3c) and portal and periportal hepatitis (Fig. 3a, b).

**Fig. 4.**
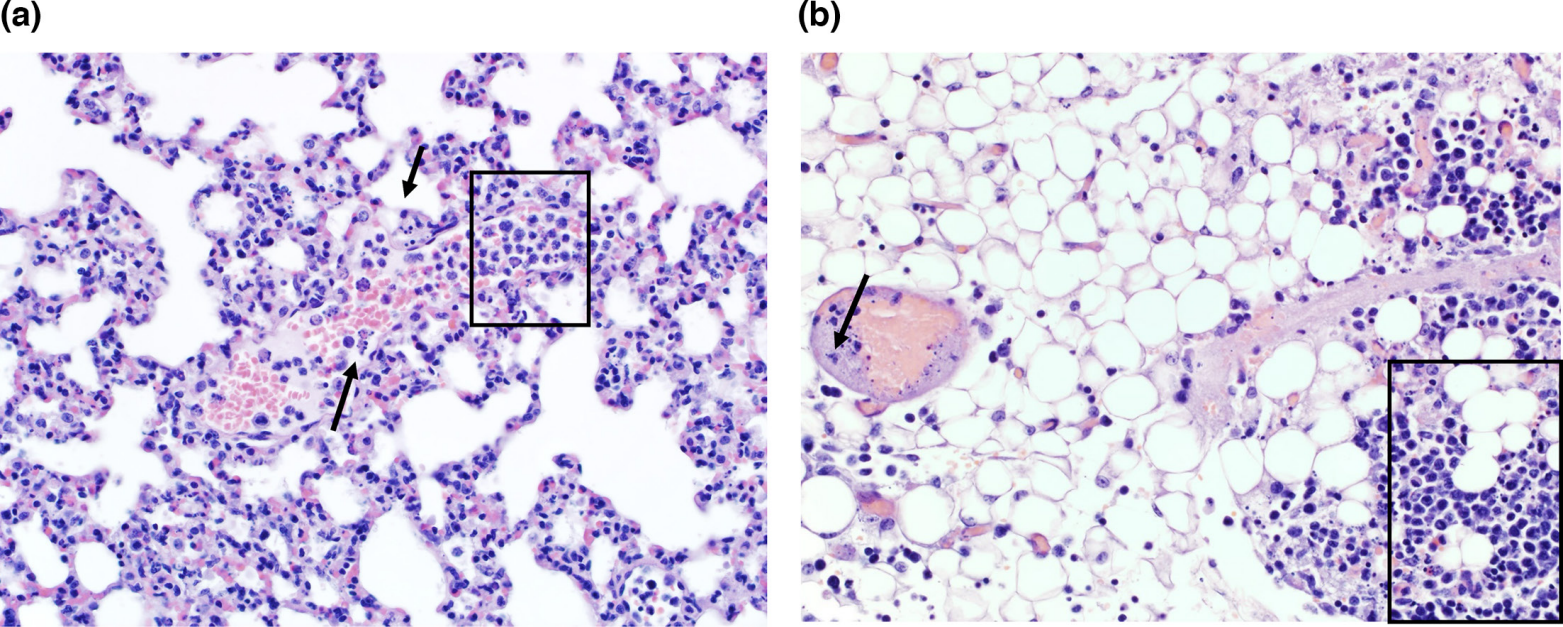
Histologic lesions of the lung of BUNV-infected IRF3/7 mouse (mouse 381). (a) Lung intravascular leukocytosis and leucocyte death (framed, 40× HE M5). (b) Lung mediastinum steatitis (framed) and vascular necrosis (black arrow) (40× HE M5).

In the lung, there was intravascular leukocytosis (Fig. 4a), and leukocyte cell death (Fig. 4b) and mild incipient accumulation of inflammatory cells in the lung parenchyma (framed in Fig. 4b). Additionally, there was mediastinal steatitis (framed in Fig. 4c) and vascular necrosis characterized by loss of structure of endothelial cells (black arrow in Fig. 4c).

**Fig. 5.**
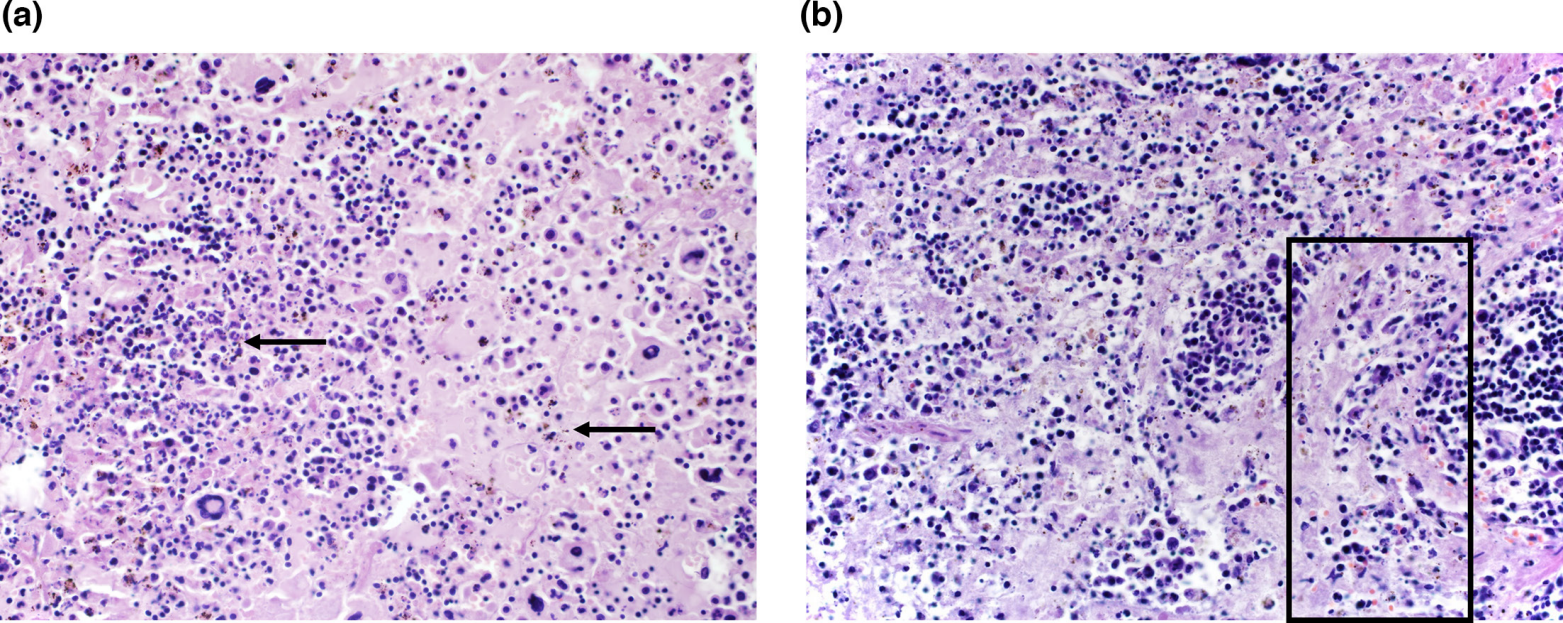
Histologic lesions include necrosis in IRF 3/7 DKO mouse. (a) Bone marrow necrosis indicated by black arrows (mouse 382, 40× HE). (b) Spleen perifollicular pulp severe necrosis indicated by framing (mouse 382, 40× HE).

**Fig. 6.**
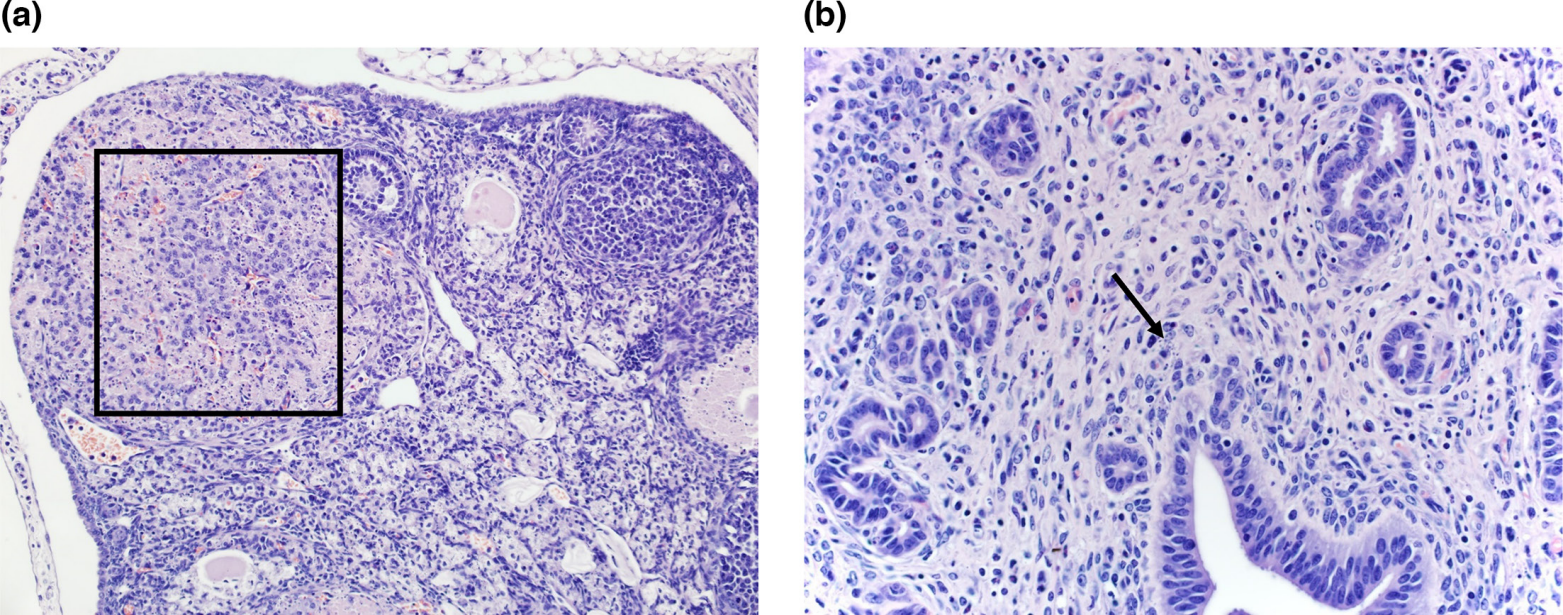
Histologic lesions of the reproductive system (mouse 381). (a) Ovary necrotizing oophoritis (framed, 20× HE M5). (b) Uterus mild necrosis of the endometrium indicated by black arrow (40× HE M5).

The lymphoid organs were necrotic (Fig. 5a). The bone marrow was affected by multifocal necrosis and the spleen by severe perifollicular necrosis (Fig. 5b). In the reproductive system, there was multifocal necrotizing oophoritis (framed in Fig. 6a) and a mild multifocal necrosis in the endometrium (black arrows in Fig. 6b).

**Fig. 7.**
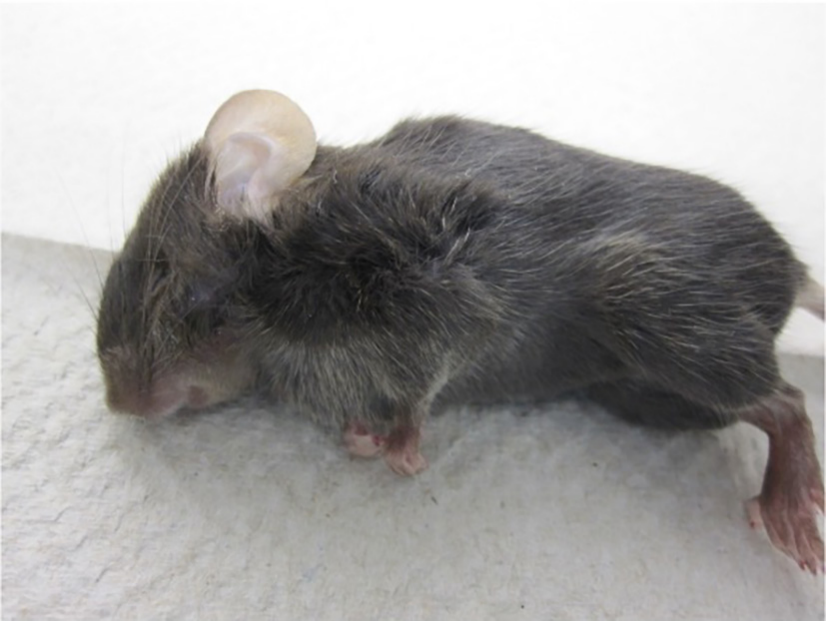
BUNV causes severe diffuse acute facial oedema in RIF 3/7 DKO mice. Representative mouse (#384) with diffuse severe facial swelling upon death at 6 dpi.

**Fig. 8.**
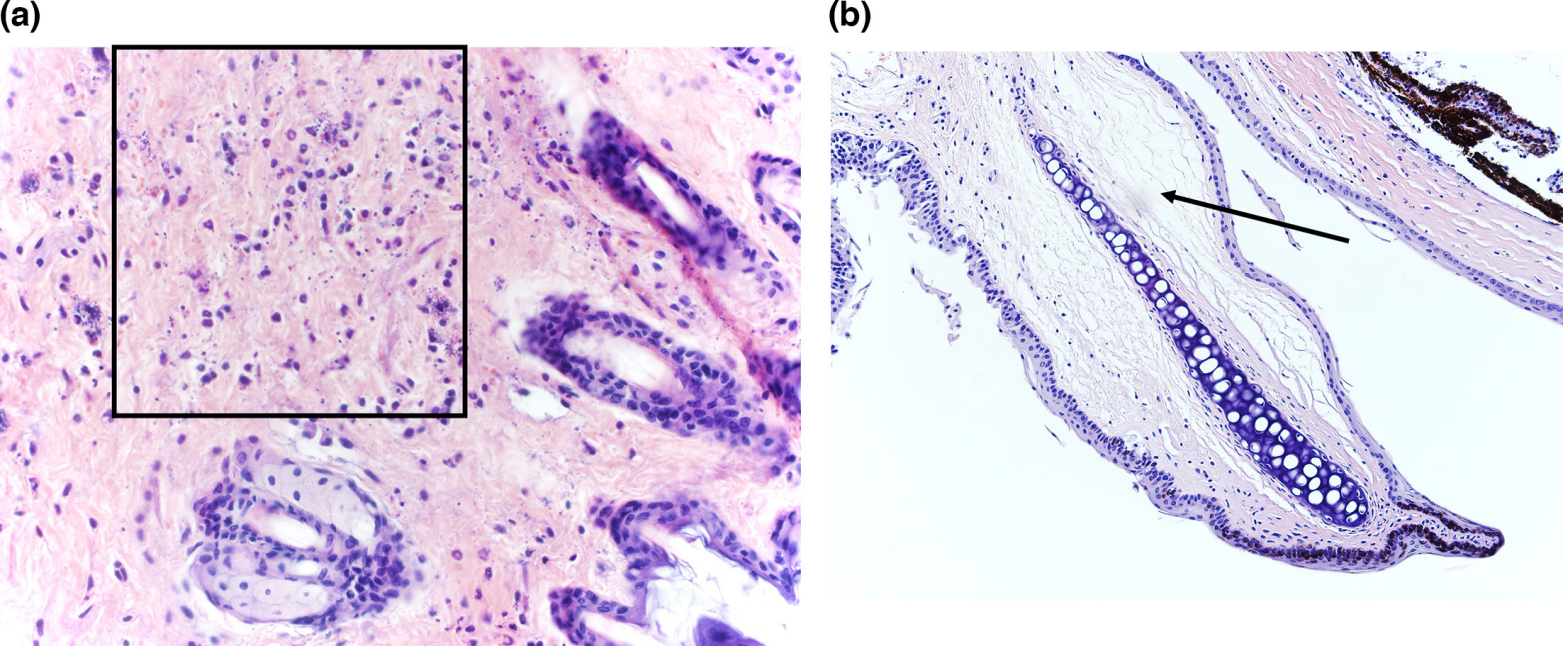
Lesions associated with facial swelling. (a) Skin necrosis and mast cell degranulation (40× HE, mouse 380). (b) Eyelid with severe diffuse oedema (20× HE, mouse 382).

The disease was also characterized by multifocal necrosis of the skin and significant facial oedema in 3/8 (37.5 %) mice (Figs7  8a). In the eyelids, there was severe diffuse acute oedema expanding the loose connective tissue and dilatation of lymphatics (Fig. 8b). These changes were responsible for the prominent facial swelling. Importantly, other viral infections in this mouse strain have not been noted to cause this particular pathology [25,32], including other Orthobunyavirus infections [33].

No significant lesions were observed in the brains, kidneys, adrenal glands, oesophagi, stomachs and intestines, skeletal muscles, bones, pancreas, thyroids or parathyroids of the mice.

## Discussion

Our observations point to the potential for a novel phenotype associated with BUNV infection in an interferon-deficient mouse model. In addition to viraemia and weight loss, we observed overt clinical signs, with major histologic lesions in three severely ill mice, mainly necrosis and inflammation. Similar lesions have been observed in an experimental study of another member of the *Orthobunyavirus* genus, Schmallenberg virus (SBV). When 0.86×10^3^ TCID50 of SBV was subcutaneously inoculated into 8-week-old type I IFN receptor knock-out (IFNAR^-/-^) mice, necropsy revealed diffusely discoloured livers, abdominal cavities containing large amounts of blood and internal bleeding into the small intestine. In histopathology, the liver showed severe hepatocellular degeneration and necrosis [34]. In strain 13, guinea pigs infected with Pichinde virus, necrotic regions of bone marrow have been described [35]. This in combination with our study provides compelling evidence for a major role for the type I IFN response in controlling *Orthobunyavirus* infections. However, our data also suggest that these viruses do have the potential to progress to severe disease, including haemorrhagic manifestations, especially in immunocompromised individuals.

Facial oedema is a specific manifestation of LUJV and LASV, which share temporal and spatial distributions with BUNV [36,41]. Facial swelling is among the most common and hallmark symptoms of LASV and LUJV and has been suggested as a discriminating symptom between Old vs. New World arenaviruses [39]. Additionally, reproductive disease has been documented, such as orchitis with severe scrotal oedema, abortions and vaginal bleeding [39,41, 42]. Postmortem evaluations of LASV-confirmed human cases showed ovaries and placentas with positively staining LASV antigens [37]. High titres of virus have been documented in female reproductive organs and placentas, suggesting a preferred site of viral replication [42,43]. Currently, this is the only non-human primate models exist that afford the opportunity to study such phenomenon histologically, and while several rodent models have been proposed for LASV, none of them describe facial oedema or reproductive disease [44]. Several other parallels can be drawn from our understanding of LASV pathology from current animal models and human infections to the BUNV phenotype described here. The liver involvement is frequently reported, and its pathology is well documented. Autopsies of individuals infected with LASV have shown hepatocellular necrosis with both individual necrotic hepatocytes and multifocal, centrilobular hepatic necrosis [43,45]. Animal models have shown a more varied spectrum of severity yet consistently show hepatocellular necrosis as well with LASV infection [44]. Other notable lesions occur in the lymphoid tissues and lungs. Splenic necrosis is a common finding in human autopsy reports as well as in several animal models [43,44]. While no report could be found concerning haemoabdomen in LASV or LUJV infections, several haemorrhagic complications and potential disseminated intravascular coagulopathy have been noted in human and animal infections of LASV and LUJV [39,41,44]. While several models have been proposed for LASV, these models generally either develop mild disease inadequate for modelling severe LASV infection or require intensive and expensive laboratory practices limiting broad accessibility [44]. Thus, the IRF3/7 DKO mouse-BUNV infection system, which produces this clinical phenotype, may serve as a surrogate model to study vascular permeability and reproductive disease, especially for these arenaviruses, at BSL-2.

Our study is not without limitations. We infected only female mice, while some studies have demonstrated a sex difference in bunyavirus infections [46]. Furthermore, additional pathology such as viral load in organs and immunohistochemistry was not available for this study, but our data point to the need for future in-depth investigations into viral tropisms and correlations with outcomes. While we did not note significant brain involvement, BUNV has been associated with neurological disease in humans and horses and experimentally in hamsters [4,47]. However, this is not a commonly reported manifestation of African strains of BUNV, while it is associated with the North American strain, Cache Valley fever [1,47].

Other arboviruses of African origin have surprised us with ‘novel’ manifestations (e.g. Zika) that have led to devastating effects [48]. Furthermore, the current outbreak of another Orthobunyavirus, the Oropouche has resulted in the manifestation of severe disease on an unexpectedly large scale, with suspected associated teratogenic effects [49]. This all points to the need to understand the spectrum of disease prior to outbreaks, and continued study of BUNV and Orthobunyaviruses in general are necessary to make sure we understand and are prepared to respond to the outbreaks of disease as the risk of arboviruses continues to expand [50,51]. Our data suggest two things: first, there is the possibility that this system of BUNV in IRF 3/7 DKO mice could act as a model system to study bunyavirus haemorrhagic disease processes at a BSL2 level, whereas many viruses are BSL3 (e.g. Ngari, Rift Valley fever) or BSL4 (e.g. Lassa, Lujo) and several are category A pathogens; second, although BUNV infection has not been associated with haemorrhagic fever in human populations, the status of this virus as a neglected tropical disease itself indicates that we likely do not fully appreciate its true potential to cause more severe disease than mild febrile illness.

## supplementary material

10.1099/jgv.0.002040 Uncited Fig. S1.

## Abbreviations

BHK: baby hamster kidney
BUNV: Bunyamwera virus
SBV: Schmallenberg virus
SM: suckling mice
