# Supplementary material for: Preliminary evidence that Bunyamwera virus causes severe disease characterized by systemic vascular and multiorgan necrosis in an immunocompromised mouse model
Source: J Gen Virol. 2024 Nov 7;105(11):002040. doi: 10.1099/jgv.0.002040 (PMC11539936; doi:10.1099/jgv.0.002040)

Supplemental Information for **Dutuze, et al.** ***BUNV pathogenesis in IRF3/7 DKO mouse***

Supplemental Figure S1: Individual viremia measures per mouse following infection with BUNV as measured by q-RT-PCR of the M segment. This demonstrates replication of the virus as the infectious titer delivered was 10^3 PFU/mL and all mice achieved higher titers.

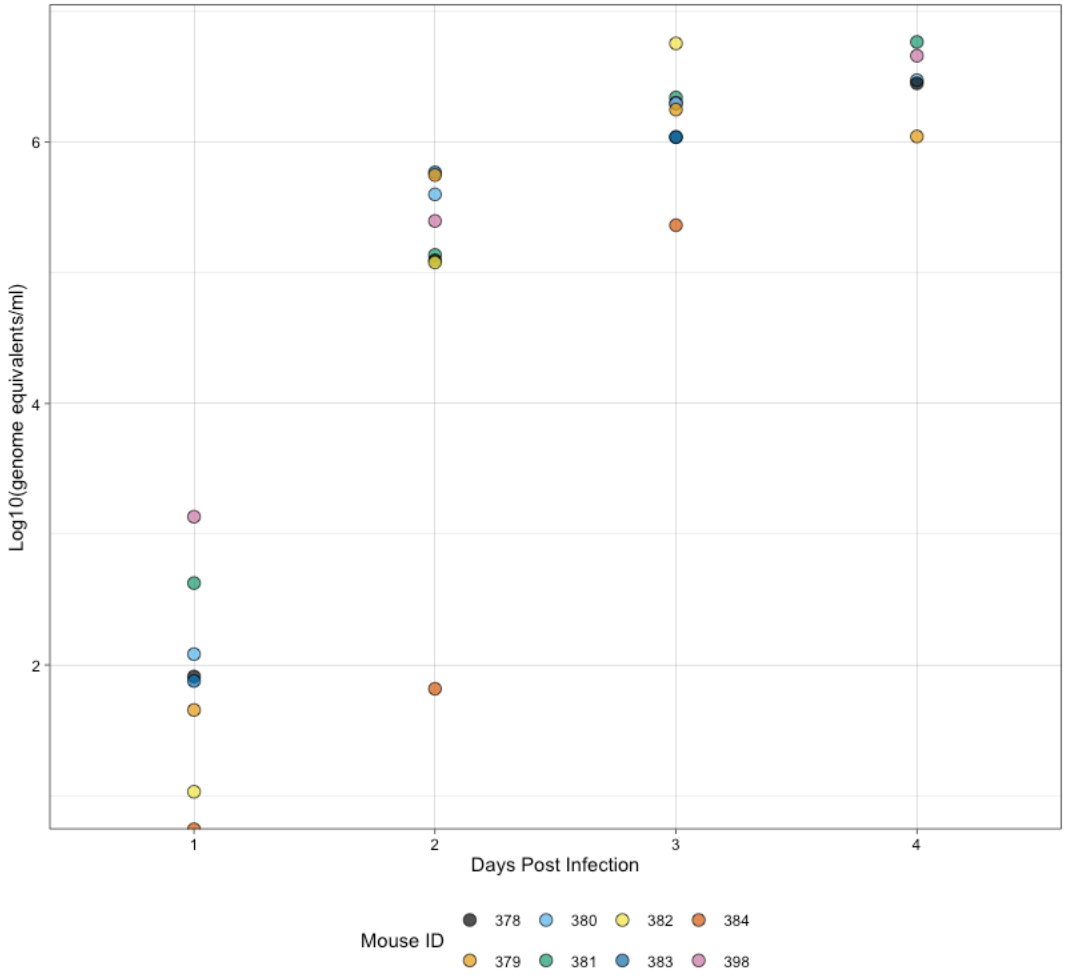

Supplemental Figure S2: Individual measures of weight (in grams) per mouse following infection with BUNV demonstrates a clinical sign of infection in all individuals.

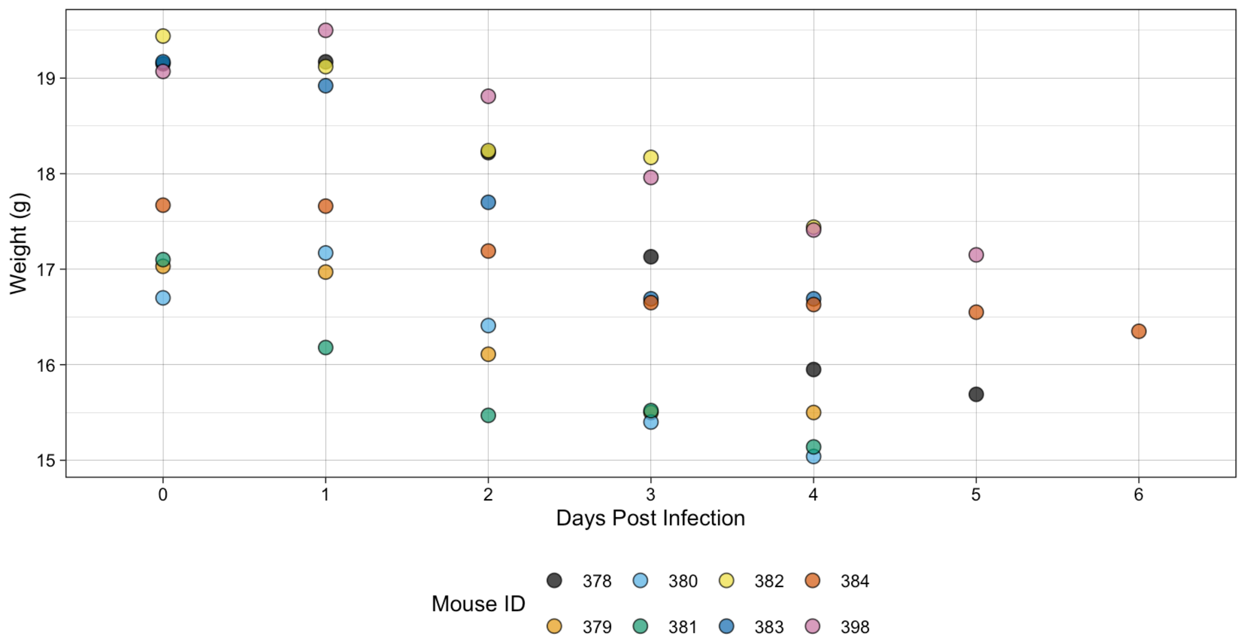

Supplement: Uncited Fig. S1. [file jgv-105-02040-s001.pdf]
